# Retirement Without God: Activism and Community Among Secular People Over 60

**DOI:** 10.1093/geroni/igaa057.1426

**Published:** 2020-12-16

**Authors:** Joseph Blankholm

**Affiliations:** University of California, Santa Barbara, Santa Barbara, California, United States

## Abstract

There are more than 1,400 nonbeliever communities in the United States and well over a dozen organizations that advocate for secular people on the national level. Together, these local and national groups comprise a social movement that includes atheists, agnostics, humanists, freethinkers, and other kinds of nonbelievers. Despite the fact that retired people over 60 dedicate most of the money and energy needed to run these groups, the increasingly vast literature on secular people and secularism has paid them almost no attention. Relying on more than one hundred interviews (including dozens with people over 60), several years of ethnographic research, and a survey of organized nonbelievers, this paper demonstrates the crucial role that people over 60 play in the American secular movement today. It also considers the reasons older adults are so important to these groups, the challenges they face in trying to recruit younger members and combat stereotypes about aging leadership, and generational differences that structure how various types of nonbeliever groups look and feel. This paper reframes scholarly understandings of very secular Americans by focusing on people over 60 and charts a new path in secular studies.

